# Comparison of the blood immune repertoire with clinical features in chronic lymphocytic leukemia patients treated with chemoimmunotherapy or ibrutinib

**DOI:** 10.3389/fonc.2023.1302038

**Published:** 2023-12-04

**Authors:** Baustin M. Welch, Bryce A. Manso, Kimberly A. Gwin, Petra K. Lothert, Sameer A. Parikh, Neil E. Kay, Kay L. Medina

**Affiliations:** ^1^ Department of Immunology, Mayo Clinic, Rochester, MN, United States; ^2^ Mayo Clinic Graduate School of Biomedical Sciences, Mayo Clinic, Rochester, MN, United States; ^3^ Department of Biomolecular Engineering, University of California, Santa Cruz, Santa Cruz, CA, United States; ^4^ Division of Hematology, Mayo Clinic, Rochester, MN, United States

**Keywords:** leukemia, ibrutinib, CLL, immune repertoire, chemoimmunotherapy

## Abstract

Chronic lymphocytic leukemia (CLL) is characterized by the accumulation of CD19^+^ CD5^+^ clonal B lymphocytes in the blood, bone marrow, and peripheral lymphoid organs. Treatment options for patients range from historical chemoimmunotherapy (CIT) to small molecule inhibitors targeting pro-survival pathways in leukemic B cells, such as the Bruton’s tyrosine kinase inhibitor ibrutinib (IBR). Using biobanked blood samples obtained pre-therapy and at standard response evaluation timepoints, we performed an in-depth evaluation of the blood innate and adaptive immune compartments between pentostatin-based CIT and IBR and looked for correlations with clinical sequelae. CD4^+^ conventional T cells and CD8^+^ cytotoxic T cells responded similarly to CIT and IBR, although exhaustion status differed. Both treatments dramatically increased the prevalence and functional status of monocyte, dendritic cell, and natural killer cell subsets. As expected, both regimens reduced clonal B cell levels however, we observed no substantial recovery of normal B cells. Although improvements in most immune subsets were observed with CIT and IBR at response evaluation, both patient groups remained susceptible to infections and secondary malignancies during the study.

## Introduction

Chronic lymphocytic leukemia (CLL) is a B cell malignancy characterized by the accumulation of clonal CD19^+^ CD5^+^ malignant B cells in the blood, bone marrow, and secondary lymphoid organs (1). Aside from lymphocytosis by the expanding CLL clone, higher rates of infection and secondary cancers are linked to a global immunodeficiency observed in CLL patients, even at early stages of disease (2, 3). Many CLL patients present with recurrent bacterial infections, which are a leading cause of morbidity and mortality among untreated and treated patients, indicating that the CLL disease process reduces immune function. Individuals with CLL also possess a higher risk of developing secondary and more aggressive malignancies (2, 4).

Chemotherapy in conjunction with anti-CD20 monoclonal antibodies, termed chemoimmunotherapy (CIT), is highly effective in reducing CLL B cell numbers along with generating excellent overall responses and complete responses and was the standard of care for untreated, young, and fit patients until recent times (5). However, anti-CD20 agents included in CIT treatment regimens were associated with increased risk of infection, including reactivation of latent infections (6). Fludarabine-based CIT is not well tolerated in older, frail patients due to associated hematologic toxicities, other malignancies, and the development of bone marrow-based malignancies such as myelodysplastic syndrome or acute myeloid leukemia (5, 7). We previously reported that pentostatin-based CIT was efficacious in young and old CLL patients and not associated with excessive bone marrow toxicity, serious infection or second malignancies seen with fludarabine based CIT (8). To our knowledge, there have been no studies detailing the impact of pentostatin-based CIT on modulation of the global immune landscape in CLL.

Treatment options for progressing patients have moved beyond CIT to the near-universal use of novel agents that target pro-survival signaling pathways in B cells based on both responses and a more tolerable toxicity profile (9, 10). One major small molecule inhibitor that initially dominated the novel agent landscape was ibrutinib (IBR), a novel agent that is a covalent inhibitor of Bruton’s tyrosine kinase (BTKi) (11). The efficacy of IBR in CLL has led to a paradigm shift in treatment bolstered by the results of multiple clinical trials. However, IBR has been demonstrated to modulate immune competency, ranging from beneficial to unfavorable (12–15). Although treatment regimens have clearly improved, CLL remains incurable.

To determine the impact on the immune repertoire of BTKi and pentostatin-based CIT (henceforth referred to as CIT), we conducted comprehensive immunophenotyping of baseline (BL) and response evaluation (RE) timepoints for both therapies. To accomplish this goal, we conducted dynamic immune profiling of T cells, monocytes, natural killer cells (NK), dendritic cells (DCs), normal B cells, and the leukemic clone, in CLL patients who received CIT and IBR treatment. An additional rationale for comparing pentostatin-based CIT is that the regimen has less cytotoxic impact on the bone marrow making it a more apt comparator to IBR (8). Importantly, we report our findings in conjunction with timely clinical correlates to gain deeper insight into the impact of therapy-associated immune alterations on CLL patient health status.

## Materials and methods

### Patients

Age-matched healthy control (HC, n=8) and CLL patient cryopreserved blood samples were provided by the Mayo Clinic CLL Tissue Bank. All patients selected for this limited study had signed informed consent to provide sequential biobanked BL and RE research samples. The RE samples for the CIT cohort were studied at the 6 month timepoint as that is the usual timeframe for evaluation of response levels post therapy initiation of CIT while the IRB treated CLL cohort are studied at the 12 and 24 month timepoints as the latter agent is known to have more delayed response in CLL patients. **Table 1** details the CIT (n=10), frontline (FL) IBR (n=5), and relapsed/refractory-CLL ibrutinib (R/R IBR, n=18) patient characteristics utilized in this comprehensive study. Cryopreserved blood samples were previously subjected to density gradient separation, eliminating granulocytes, red blood cells, and platelets, and the cells were retained on ice post-thaw before flow staining. Paired BL and RE patient samples were processed simultaneously to limit variability that might be imposed by sample handling. All patient samples were divided into blinded, randomized batches that had representation of all CLL treatment cohorts present within each batch in order to limit any batch effects from confounding data analysis.

**Table 1:** CLL patient disease characteristics and prognostic indicators.

Patient ID*^a^*	Group*^b^*	Treatment*^c^*	Age	Sex	IGHV status	VH Family*^d^*	Percent mutation*^d^*	CD49d*^d^*	FISH status at BL*^e^*	Rai stage at BL	CLL-IPI score*^d,f^*
**CLL 1**	CIT	PCO	58.0	F	UM	3-33	0.0	Neg	Del(13q)	0	2
**CLL 2**	CIT	PCO	75.4	M	UM	1-69	0.0	Pos	Trisomy 12	II	6
**CLL 3**	CIT	PCO	63.8	F	M	4-61	9.5	Pos	Trisomy 12	II	3
**CLL 4**	CIT	PCO	52.9	F	UM	1-3	0	Neg	Other	I	3
**CLL 5**	CIT	EGCG, PCO	57.3	M	M	3-23	5	Neg	Normal	0	0
**CLL 6**	CIT	EGCG, PCO	57.3	M	M	4-59	23.1	Neg	Del(13q)	I	0
**CLL 7**	CIT	PCO	72.8	M	UM			Pos	Del(11q)	0	5
**CLL 8**	CIT	EGCG, PCO	64.9	M	M	4-59	5.5	Neg	Trisomy 12	I	1
**CLL 9**	CIT	PCO	77.9	M	UM			Pos	Trisomy 12	I	4
**CLL 10**	CIT	PCO	57.8	M	UM	3-11	0.4	Neg	Del(13q)	II	3
**CLL 11**	FL IBR	IBR	48.6	M	UM				Del(17p)	II	7
**CLL 12**	FL IBR	IBR	58.0	M	UM	3-30	0		Normal	II	3
**CLL 13**	FL IBR	IBR	77.5	F	M	3-11	2.9		Del(13q)	II	2
**CLL 14**	FL IBR	IBR	59.9	M	UM			Pos	Trisomy 12	III	2
**CLL 15**	FL IBR	IBR	68.3	F	UM			Neg	Del(13q)	I	4
**CLL 16**	R/R IBR	IBR	60.4	M	UM	3-11	0.0	Neg	Del(13q)	IV	
**CLL 17**	R/R IBR	IBR	79.9	F	UM	1-18	0.0	Pos	Del(13q)	0	5
**CLL 18**	R/R IBR	IBR	66.6	M	UM	1-8	0.0	Neg	Del(13q)	IV	5
**CLL 19**	R/R IBR	IBR	81.0	M	M	2-5	5.1	Pos	Trisomy 12	IV	2
**CLL 20**	R/R IBR	IBR	76.0	M	M	3-21	4.6	Pos	Del(13q)	IV	3
**CLL 21**	R/R IBR	IBR	85.5	F	UM	1-69	0.0	Pos	Trisomy 12	II	2
**CLL 22**	R/R IBR	IBR	62.5	M	UM	3-20	0.4	Neg	Del(11q)	I	
**CLL 23**	R/R IBR	IBR	76.0	M	UM	4-31	0.0	Pos	Trisomy 12	I	6
**CLL 24**	R/R IBR	IBR	49.3	M	UM	7-4-1	0.0	Neg	Del(13q)	II	3
**CLL 25**	R/R IBR	IBR	77.6	M	M	3-11	2.9	Neg	Normal	I	2
**CLL 26**	R/R IBR	IBR	73.3	F	M	3-53	3.0	Pos	Other	IV	2
**CLL 27**	R/R IBR	IBR	58.8	M	UM	4-39	0.0	Pos	Trisomy 12	IV	3
**CLL 28**	R/R IBR	IBR	74.2	F	UM	2-70	1.9	Neg	Del(11q)	I	2
**CLL 29**	R/R IBR	IBR	56.3	M	UM				Del(11q)	I	
**CLL 30**	R/R IBR	IBR	73.6	M	M	6-1	5.0	Pos	Del(11q)	II	4
**CLL 31**	R/R IBR	IBR	70.6	M	M	5-51	6.8	Neg	Del(13q)	III	0
**CLL 32**	R/R IBR	IBR	88.0	F	UM	1-69	0.0	Neg	Normal	IV	6
**CLL 33**	R/R IBR	IBR	73.7	M	UM	1-2	0.4	Neg	Del(13q)	II	3

(A) All patients except for individual CLL-33 had a positive test result for β2M. (B) “Baseline” for “R/R IBR” Tx group indicates patient evaluation timepoint prior to ibrutinib treatment but post-previous treatment regimens. (C) PCO indicates pentostatin, cyclophosphamide, and ofatumumab treatment regimen. EGCG indicates epigallocatechin. (D) Empty cells indicate “data not available”. (E) Fluorescence in situ hybridization (FISH). (F) CLL International Prognostic Index (CLL-IPI).

### Flow cytometry

Thawed blood samples were incubated with an Fc blocking antibody (Miltenyi Biotec, San Diego, CA, USA) on ice for 30 minutes followed by incubation with fixable viability dye and monoclonal antibody cocktails (**Supplementary Table 1**) on ice for an additional 30 minutes (protected from light). Equivalent numbers of cells were stained for all flow analyses. Following washing with 1X PBS, the cells were resuspended in 1% paraformaldehyde (Electron Microscopy Sciences, Hatfield, PA, USA) and kept refrigerated in the dark until sample acquisition. Flow cytometry data was acquired on a LSRFortessa X-20 (BD Biosciences, Franklin Lakes, NJ, USA) that was standardized to allow for the comparison of direct geometric mean fluorescent intensity (gMFI) across experiments using a modified protocol outlined by Perfetto et al. and Manso et al. (16–18).

### Flow cytometry analysis

FlowJo 10.5.3 (Becton Dickinson) was used for the analysis of flow cytometry.fcs file data. Gating strategies for each immune cell panel (**Supplementary Table 1**) are shown in **Supplementary Figures 2**, **4**-**6**. When analyzing leukocyte populations in blood we report cellular populations as a percent of CD19^+^ CD5^+^-excluded PBMC (HC and CLL) as described in Manso et al. (19). Gating trees are shown in contour format. After backgating from total nucleated cells and lymphocytes, positive gates were set and applied to the indicated cellular populations. Gates were batch-corrected, minimizing variation between experiments to accurately capture populations of interest.

### Statistical analysis

**Figure 1:**
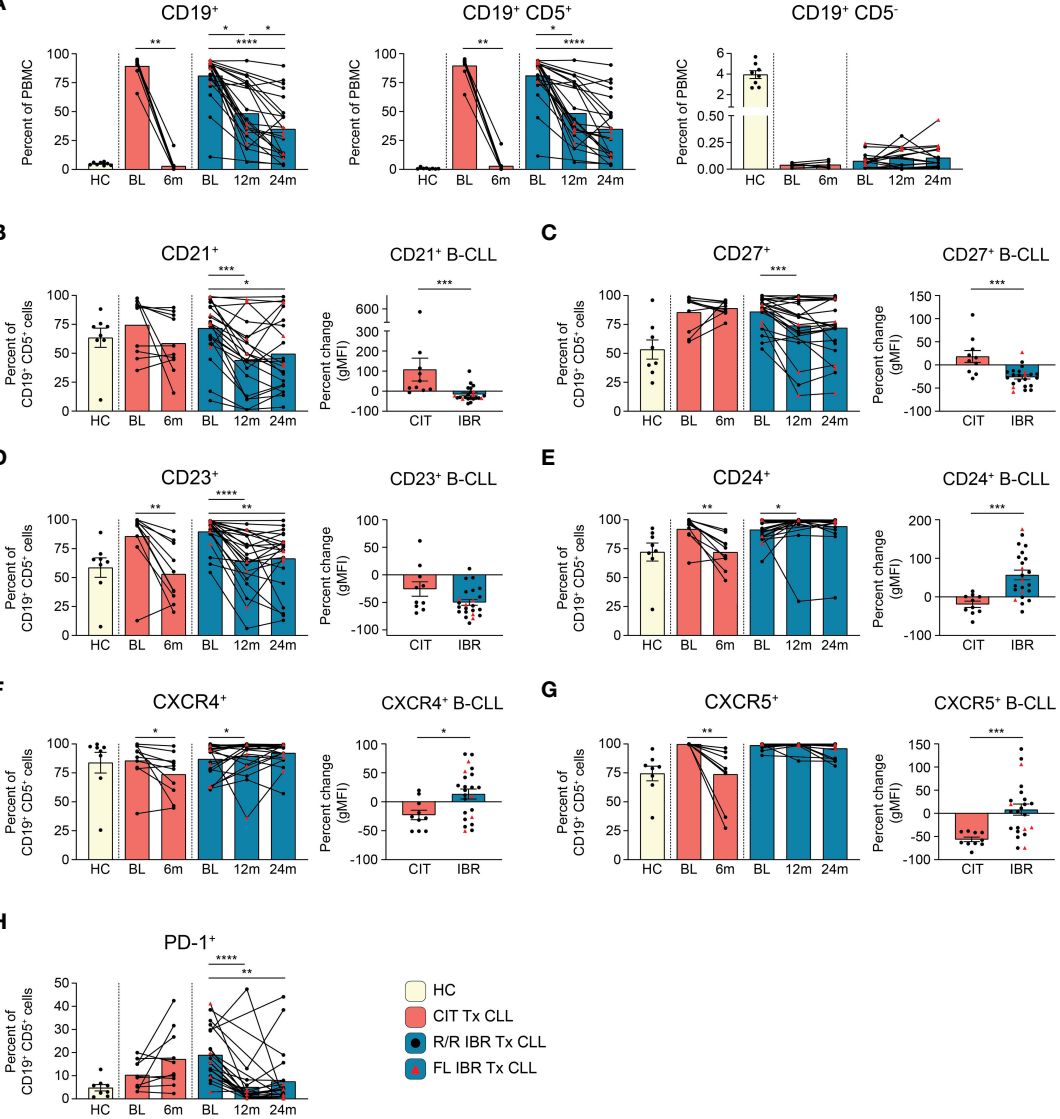
B cell population and surface protein expression dynamics during CLL treatment. **(A)** Frequencies of CD19^+^, CD19^+^ CD5^+^, and CD19^+^ CD5^-^ cells are reported as a percent of PBMC. **(B–G)** The frequency of CD21^+^, CD23^+^, CD24^+^, CD27^+^, CXCR4^+^, or CXCR5^+^ CD19^+^ CD5^+^ B cells from HC and CLL patient groups is reported as a percent of CD19^+^ CD5^+^ B cells. The percent change in the gMFI between BL and RE of indicated treatment cohort is reported for surface proteins on the indicated surface marker expressing CD19^+^ CD5^+^ cells. **(H)** Frequency of PD-1^+^ cells as a percent of CD19^+^ CD5^+^ cells from HC and CLL patient groups. **(A–H)** Statistical significance shown are as follows *p<0.05, **p<0.01, ***p<0.001, and ****p<0.0001.

**Figure 2:**
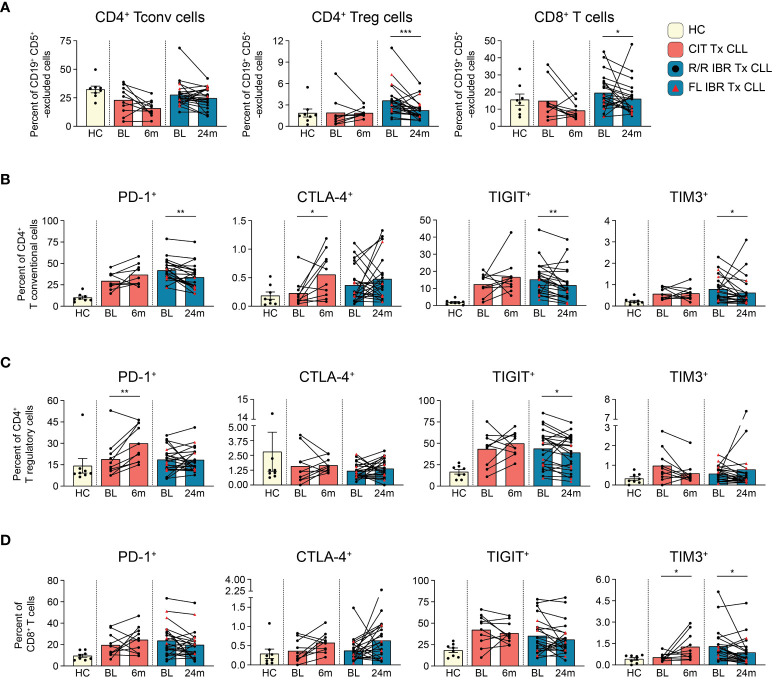
T cell subset dynamics in response to CLL treatment. **(A)** Frequency of CD4^+^ T conventional, CD4^+^ T regulatory, and CD8^+^ T cells are reported as a percent of CD19^+^ CD5^+^ -excluded cells. **(B)** Frequency of PD-1^+^, CTLA-4^+^, TIGIT^+^, TIM3^+^ CD4^+^ T conventional cells reported as a percent of CD4^+^ T conventional cells. **(C)** Frequency of PD-1^+^, CTLA-4^+^, TIGIT^+^, TIM3^+^ CD4^+^ T regulatory cells reported as a percent of CD4^+^ T regulatory cells. **(D)** Frequency of PD-1^+^, CTLA-4^+^, TIGIT^+^, TIM3^+^ CD8^+^ T cells reported as a percent of CD8^+^ T cells. **(A–D)** Statistical significance shown are as follows *p<0.05, **p<0.01, and ***p<0.001.

**Figure 3:**
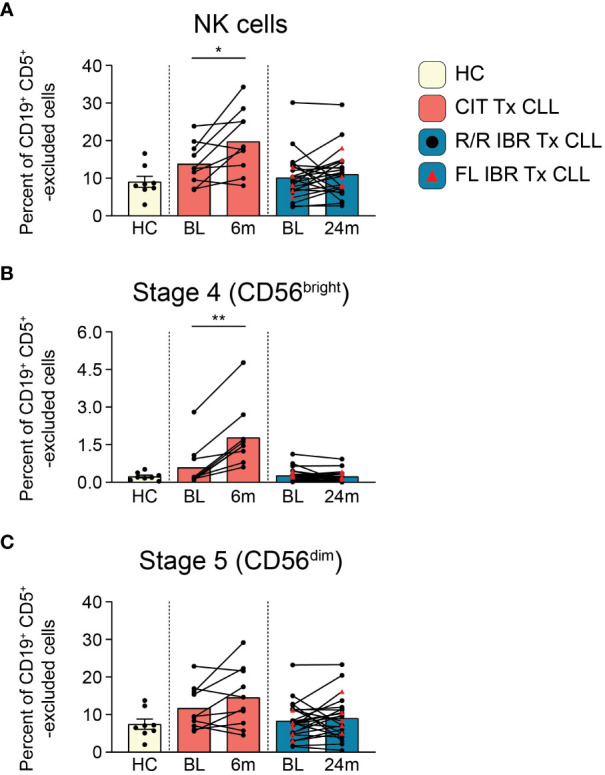
NK cell population dynamics during CLL treatment. **(A)** Frequency of NK cells reported as a percent of CD19^+^ CD5^+^ -excluded cells. **(B)** Frequency of stage 4 CD56^bright^ NK cells reported as a percent of CD19^+^ CD5^+^ -excluded cells. **(C)** Stage 5 CD56^dim^ NK cells reported as a percent of CD19^+^ CD5^+^ -excluded cells. **(A–C)** Statistical significance shown are as follows *p<0.05 and **p<0.01.

**Figure 4:**
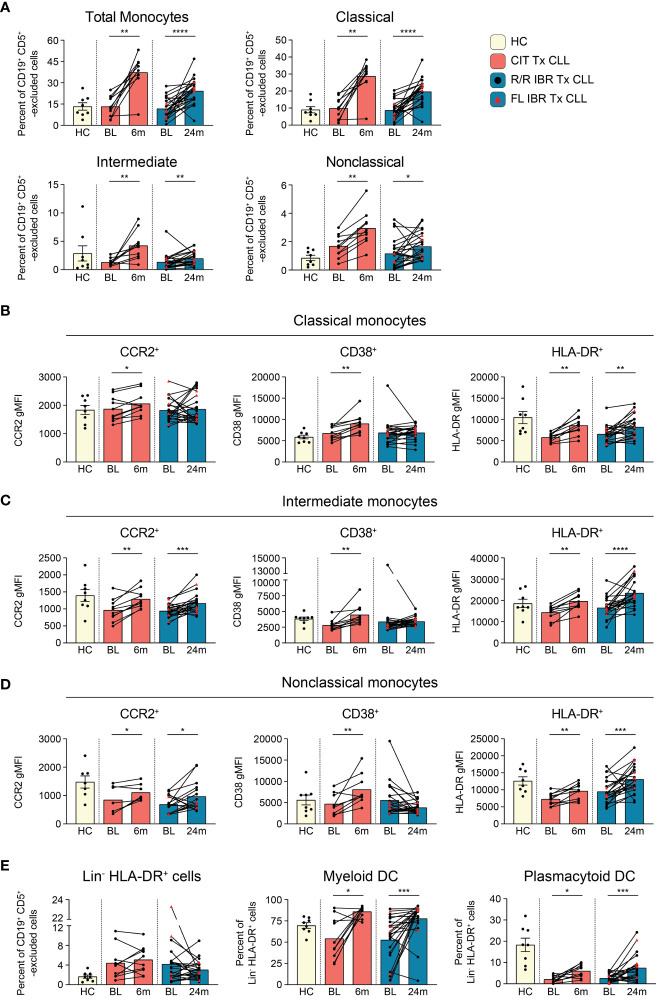
Monocyte subset population dynamics during CLL treatment. **(A)** Frequency of total (CD66b^-^ CD3e^-^ CD19^-^ CD5^-^ monocyte side scatter), classical (CD14^+^ CD16^-^), intermediate (CD14^+^ CD16^+^), and nonclassical (CD14^-^ CD16^+^) monocytes as a percent of CD19^+^ CD5^+^ -excluded cells. **(B)** gMFI for CCR2, CD38, and HLA-DR is reported for indicated surface marker expressing classical monocytes. **(C)** gMFI for CCR2, CD38, and HLA-DR is reported for indicated surface marker expressing intermediate monocytes **(D)** gMFI for CCR2, CD38, and HLA-DR is reported for indicated surface marker expressing nonclassical monocytes. **(E)** Frequency of Lin^-^ (CD3^-^ CD5^lo/-^ CD14^-^ CD19^-^ CD20^-^ CD56^-^) HLA-DR^+^ cells reported as a percent of CD19^+^ CD5^+^ -excluded cells. The frequency of myeloid and plasmacytoid DCs reported as a percent of Lin^-^ HLA-DR^+^ cells. **(A–E)** Statistical significance shown are as follows *p<0.05, **p<0.01, ***p<0.001, and ****p<0.0001.

Statistical tests are indicated in all figure legends and were performed using GraphPad Prism version 9.1.0 (GraphPad Software, San Diego, CA, USA). Three sets of statistical analyses were applied to determine significance between different groups. When comparing BL to 6-month RE in the CIT treated cohort or BL to 24-month RE in the IBR treated cohort, the Wilcoxon matched-pair signed rank test was utilized (shown in relevant figures). To analyze the IBR treated cohort, which includes a 12-month RE timepoint, Dunn’s multiple comparisons test was used. Given this, we omitted 4 of the 23 IBR treated patients, as they did not have a 12-month RE sample. This data is shown in **Figure 1** and **Supplementary Figure 1**. To confirm that the omission of 4/23 IBR treated CLL patients did not affect significance of the paired analysis we also utilized the Wilcoxon matched-pair signed rank test strictly comparing BL to 24-month RE containing all 23 IBR treated CLL patients (shown in **Figures 2**-**4**). When making comparisons of parameters to HC, we utilized Dunn’s multiple comparisons test comparing either HC, CIT BL, and 6-month RE or HC, IBR BL, 12-month, and 24-month RE. Where discussed, p-values are inserted in the results section. To analyze the percent change in the gMFI of the indicated surface proteins on CD19^+^ CD5^+^ B cells shown in **Figures 1B-G**, we performed Mann Whitney tests. The HC bar graphs indicate the mean standard error of the mean (SEM).

## Results

### B cell dynamics with therapy

This study utilized biobanked cryopreserved blood samples from 8 age-matched HC and 33 CLL patients that were grouped as follows: pentostatin-based CIT (n=10) and IBR [n=23; 5 had received IBR as frontline therapy and 18 in a relapsed/refractory setting (R/R)]. Relevant patient clinical CLL prognostics are provided in **Table 1**. We also compared time-to-treatment (TTT) after initial CLL diagnosis; frontline IBR treated group (1.36 ± 0.91 year TTT), R/R patients (1.89 ± 1.34 year before initiation of frontline IBR), and CIT treated patients (4.62 ± 2.71 year TTT). BL indicates sample acquisition prior to the indicated treatment. The BL timepoint for R/R patients on IBR indicates sample collection after the previous treatment regimen ended and prior to initiation of IBR therapy. The RE timepoint for the CIT cohort in this study was 6 months while the RE for the FL or R/R IBR cohorts were 12 and/or 24 months. As stated above but to reinforce the evaluation timepoints the latter timepoints were chosen as most mature novel agent based clinical trials show continuing clinical responses for CLL patients at least up to two years (10).

CLL (CD19^+^ CD5^+^) cellular dynamics were evaluated with the two therapies (**Figure 1A**). The flow cytometry panel used to evaluate CLL B cell phenotypic changes is shown in **Supplementary Table 1**. As expected, total CD19^+^ cells were significantly elevated at BL in untreated and R/R patients compared to HC (HC vs CIT BL p= 0.0311 and HC vs IBR BL p= <0.0001) (**Figure 1A**, left panel). CD19^+^ B cells were further subdivided into CD19^+^ CD5^+^ and CD19^+^ CD5^-^ B cells (**Supplementary Figure 2**). In CLL patients, CD19^+^ cells were largely, and as expected, CD19^+^ CD5^+^ light chain restricted (either Ig k^+^ or Ig k^-^, **Figure 1A**, middle panel and **Supplementary Figure 2C**) whereas in age-matched HC the frequency of CD19^+^ CD5^+^ was less than 1.0% (0.83 ± 0.67% - range 0.24%-2.37%) of total PBMC (**Figure 1A**, middle panel and **Supplementary Figure 3A**) and polyclonal (**Supplementary Figure 2B**). CIT largely ablated CLL B cells at the 6-month RE timepoint, whereas CLL frequencies at the 12- and 24- month RE timepoints with IBR treatment were heterogeneous (**Figure 1A**, middle panel). We note that Absolute Lymphocyte Counts (ALCs) in both CIT and IBR patients were in the normal range at RE (**Supplementary Figure 1A**). Importantly, CD19^+^ CD5^-^ conventional B cell frequencies did not substantially recover to HC levels at RE timepoints studied for either treatment group (**Figure 1A**, right panel).

Next, we examined alterations in cell surface markers relevant to B cell function, including roles in signaling, maturation, costimulation, and migration (**Figure 1B**, **Supplementary Figures 3B, C**). Alterations in surface expression were determined by percent change of geometric mean fluorescence intensity (gMFI) of CLL B cells expressing the indicated surface protein at RE (CIT: 6-months, IBR: 24-months) compared to BL. Provided for each marker is the frequency range of CD19^+^ CD5^+^ cells in HC expressing the marker.

CD21 is a costimulatory molecule integrated with the B cell coreceptor complex that binds complement bound to pathogens/antigens (20). At 12- or 24-months RE, we observed reductions in frequencies of CLL cells expressing CD21 and noted significantly reduced surface expression (gMFI) in the IBR cohort. In contrast this difference was not observed on the majority of CLL cells in CIT treated patients (**Figure 1B**).

CD27 is a costimulatory molecule that is commonly utilized as a marker of memory B cells. The marker is expressed by most CLL B cells, where it functions to mediate adherence to stromal cells (21, 22). Frequencies and expression levels were decreased in most IBR treated patients, consistent with previous reports (**Figure 1C**) (23–26). These attributes were variable in CLL cells in CIT patients, like CD21.

CD23 is the Fc receptor for IgE, a known negative regulator of BCR signaling, and is commonly used to phenotype CLL cells (27, 28). We confirmed high expression of CD23 at BL in CLL cells compared to HC CD19^+^ CD5^+^ B cells (**Figure 1D**, **Supplementary Figure 3B**, right panel). Expression levels of CD23 on the residual CLL cells were significantly decreased at RE in both therapy groups (**Figure 1D**). Similarly, frequencies of CLL cells expressing CD23 were decreased in both therapy groups, although we note that a small number of IBR treated patients showed minimal changes in frequencies of CD23^+^ CLL cells (**Figure 1D**).

CD24 is associated with B cell maturation, and is thought to act as a modulator of B cell signaling (29). CD24 is highly expressed by most CLL cells at BL (**Figure 1E**). Frequencies of CD24^+^ CLL cells were significantly decreased from BL in the CIT treated cohort (p=0.0039). In contrast, except for a single patient, percentages and expression levels of CD24 on CLL cells were significantly increased from BL at 12-month RE in the IBR treatment cohort (**Figure 1E**, p=0.0105).

CXCR4 and CXCR5 are chemokine receptors involved in B cell migration. Both are highly expressed on CLL cells at diagnosis (**Figures 1F, G**) (30). Frequencies of CXCR4^+^ and CXCR5^+^ CLL cells decreased with CIT and had varied changes with IBR treatment, the latter more likely related to the postulated mechanisms of IBR inducing homing patterns and observed blood lymphocytosis of CLL cells (31–33).

PD-1 is a negative regulator of B cell proliferation and cytokine production in B cells (34). IBR monotherapy has been reported to induce a selective and durable decrease of the PD-1/PD-L1 pathway (35). Frequencies of PD-1^+^ CLL cells were indeed reduced upon IBR treatment over the 12- and 24-month (p<0.0001 BL vs 12-month RE and p=0.0035 BL vs 24-month RE) study period whereas with CIT they remained unchanged or increased (**Figure 1H**).

CD20 is a marker involved in the differentiation of B cells into plasma cells and low expression is frequently observed on CLL cells (36). We note that expression levels of CD20 on CLL cells is reduced compared to normal CD19^+^ CD5^+^ CD20^+^ B1 cells (**Supplementary Figure 3C**, left panel). The low expression of CD20 on CLL cells was reduced by CIT and IBR treatment (**Supplementary Figure 3C**, middle and right) (37). Similarly, we did not observe significant alterations in expression of CD19 or CD5 in either treatment group (**Supplementary Figure 3B**).

Finally, we evaluated expression of surface markers associated with poor prognosis: CD38 and CD49d. No significant alteration in frequencies or expression levels of CD38 were observed at RE in either treatment group (data not shown). CD49d is the α4 subunit of the α4β1 integrin VLA-4 and has a role in CLL migration and retention in bone marrow and lymph nodes. Importantly, CD49d is a highly relevant predictor of overall survival and progression free survival in CLL (38, 39). Notably, CD49d expression has been shown to associate with reduced lymphocytosis in IBR treated patients (40). Frequencies of CD49d^+^ CLL cells were variable at BL in both treatment groups (**Supplementary Figure 3D**). We observed no consistent change in frequencies of CD49d^+^ CLL cells in the CIT cohort at RE (**Supplementary Figure 3D**). In contrast, two opposite observations were made in the CD49d^+^ CLL patient cohort with IBR treatment (n=9). 3/9 patients had decreased frequencies of CD49d^+^ cells, while 6/9 patients maintained or had increased frequencies of CD49d^+^ cells with IBR treatment (**Supplementary Figure 3D**). To determine if the latter patient group correlated with sustained lymphocytosis, we examined if increased frequencies of CD49d^+^ cells correlated with persistent blood lymphocytosis. As shown in **Supplementary Figure 3E**, there was no bias between CD49d expression and blood observable leukemic cell burden in our study cohort.

**Figure 5:**
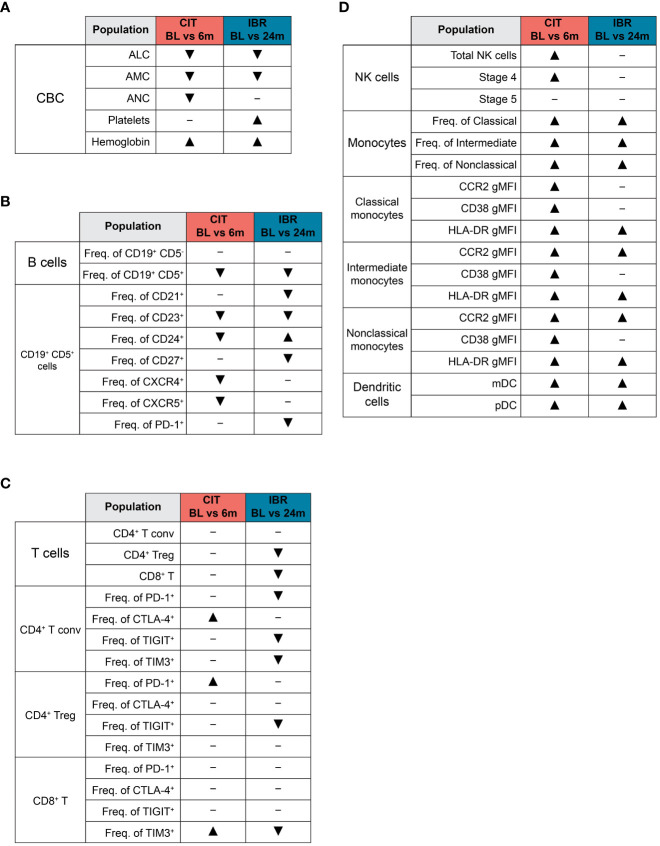
Summary of global immune population dynamics upon CLL treatment. **(A–D)** “▴” indicates a statistically significant increase. “▾” indicates a statistically significant decrease. “─” indicates no statistically significant change. **(A)** Summarizes the statistically significant changes that occur between CLL treatment cohort BL and RE timepoints in the CBC data shown in **Supplementary Figure 1**. **(B)** Summarizes the statistically significant changes that occur between CLL treatment cohort BL and RE timepoints in B cell subsets and the CLL B cell surface marker expression dynamics shown in **Figure 1**. **(C)** Summarizes the statistically significant changes that occur between CLL treatment cohort BL and RE timepoints in the dynamics of major T cell subsets and exhaustion marker surface expression shown in **Figure 2**. **(D)** Summarizes the statistically significant changes that occur between CLL treatment cohort BL and RE timepoints in innate cell subsets and monocyte functional marker surface expression shown in **Figures 3**, **4**.

Taken together, these data reveal similar and contrasting changes in CLL surface marker dynamics in response to CIT vs. IBR treatment (summarized in **Figure 5B**). Notably, neither CIT nor IBR restored normal CD19^+^ CD5^-^ conventional B cells to levels seen in HC at the response evaluation timepoints (**Figure 1A**, right panel). The deficiency in conventional B cells is consistent with the known hypogammaglobulinemia in most treated and responding CLL patients (**Supplementary Table 2**) (41, 42).

### T cell subset dynamics in response to therapy

T cells are well known to be dysfunctional in CLL patients and display features of exhaustion (25, 43–46). To document the impact of CIT and IBR treatment on T cell subset dynamics, we utilized a 12-parameter flow cytometry panel that allowed discrimination of three major T cell subsets and expression of four exhaustion markers: PD-1, CTLA-4, TIGIT, and TIM3 (**Supplementary Table 1**). First, we compared major T cell subset frequencies (gating scheme provided in **Supplementary Figure 4**) at BL and RE for CIT and IBR. For simplicity, only the 24-month RE data is shown for IBR, as we did not observe any significant differences in T cell subset frequencies between the 12- and 24-month RE timepoints. Frequencies of T conventional (CD3^+^ CD4^+^ CD25^-^), T regulatory (CD3^+^ CD4^+^ CD25^+^ CD127^-^), and T cytotoxic (CD3^+^ CD8^+^) subsets were compared after CD19^+^ CD5^+^ exclusion (**Figure 2A**) (19, 47). The same subsets in CD19^+^ CD5^+^ excluded age-matched HC are shown for reference (**Figure 2A**). CIT had variable effects on frequencies of the three T cell subsets at RE. CD4^+^ Tconv and CD8^+^ cytotoxic T cells trended down with CIT although the change did not reach significance. CD4^+^ Tconv were largely unchanged at RE compared to BL in the IBR cohort (**Figure 2A**). In contrast, significant reductions in CD4^+^ Treg and CD8^+^ T cells were seen in the IBR treated cohort (p=0.0006 and p=0.0214 BL vs 24-month RE). These data show differential effects on T cell subset dynamics upon the use of CIT versus IBR therapies (summarized in **Figure 5C**).

Next, we examined the frequency of exhaustion marker positive T cells and compared treatment associated alterations. Of the four exhaustion markers evaluated, PD-1, TIGIT and TIM3 were more abundantly expressed than CTLA-4 (**Figures 2B–D**). Frequencies of PD-1^+^ and TIGIT^+^ cells in all three T cell subsets were generally elevated at BL in both treatment cohorts compared to HC (**Figures 2B–D**). Consistent with published data, frequencies of PD-1^+^ CD4^+^ Tconv cells were significantly reduced at RE in the IBR treated cohort (46). We did not observe a further or significant decline in frequencies of PD-1^+^ Tregs or CD8^+^ cells at 12 months (data not shown) or 24 months IBR treatment (**Figures 2C, D**).

TIGIT limits adaptive and innate immune responses [reviewed in (48)]. Similar to PD-1, frequencies of TIGIT^+^ cells were increased at BL in all three T cell subsets compared to HC (Tconv p<0.0001, Treg p= 0.0059, and CD8 T p= 0.0838 HC vs BL in IBR treatment) (Tconv p= 0.0063, Treg p= 0.0183, CD8 T p= 0.0069 HC vs BL CIT treatment). Percentages of TIGIT^+^ T cells were not reduced upon CIT treatment (**Figures 2B–D**). In contrast, TIGIT^+^ Tconv and Tregs, but not CD8^+^ cells, were reduced by IBR.

TIM-3 is an inhibitory receptor shown to dampen anti-cancer T cell responses [reviewed in (49)]. TIM3^+^ frequencies were elevated at BL in CD4^+^ Tconv and CD8^+^, but not CD4^+^ Treg (**Figures 2B–D**). CIT treatment did not significantly impact TIM-3 frequencies in either CD4^+^ T cell subset but did result in increased frequencies of TIM-3^+^ CD8 cells. IBR treatment reduced frequencies of CD4^+^ Tconv and CD8^+^ cells expressing TIM-3. This finding is consistent with our recent report of IBR based therapy enhancing T cell function (50).

CTLA-4 is a negative regulator of T cell activation [reviewed in (51)]. CTLA-4 frequencies were low at BL in both patient cohorts (**Figures 2B–D**). The increased pattern of CTLA-4 expression on T cell subsets, in general, was like PD-1 and TIGIT in CIT patients, although significant only in CD4^+^ Tconv cells. Alterations in CTLA-4 expression varied considerably in the IBR cohort (**Figure 2B**).

In summary, the elevated frequencies of PD-1^+^, TIGIT^+^ and TIM3^+^ on CD4^+^ Tconv at BL were significantly reduced at RE in IBR treated patients. We note that the higher frequencies at BL were enriched in the R/R IBR patients, compared to those that received IBR frontline (**Figure 2B**). In contrast to IBR, PD-1^+^ and CTLA-4^+^ cells were increased in all 3 T cell subsets in CIT treated patients that reached significance in CD4^+^ Treg (**Figures 2B–D**). Taken together, these data show contrasting alterations in exhaustion status in T cells after CIT or IBR treatment when studied at RE and are summarized in **Figure 5C**. We note that some treated patients showed frequencies of exhaustion marker positive T cells in the HC range.

### NK population dynamics during CLL therapy

NK cells survey for the presence of abnormal cells displaying “missing-self” or “abnormal-self”. NK cell alterations in number and function have been characterized in CLL patients (52–54). We examined NK subset frequencies using the NK cell developmental pathway summarized in J. Yu, et al. (55). Total NK cell frequencies (CD66b^-^ CD14^-^ CD3e^-^ CD56^+^, gating shown in **Supplementary Figure 5**) at BL were comparable to HC in both CIT and IBR cohorts (HC vs CIT BL p=0.3965 and HC vs IBR BL p>0.9999) (**Figure 3A**). We next examined two NK cell subpopulations at BL and RE: regulatory stage 4 (CD56^bright^ CD94^+^ CD16^-^) that produce high amounts of proinflammatory cytokines and cytotoxic stage 5 (CD56^dim^ CD94^+/-^ CD16^+^) that comprise the major circulating populations (**Figures 3B, C**). Stage 4 NK cells increased significantly in CIT treated CLL, while there was no change in the IBR cohort (**Figure 3B**). We did not observe significant changes in stage 5 NK cells for either treatment group (**Figure 3C**). This comparative analysis shows a striking difference of CIT versus IBR on NK cell population dynamics (summarized in **Figure 5D**).

### Monocyte and dendritic cell population dynamics during CLL therapy

Monocytes are precursors to macrophages which provide an innate barrier to infection in tissues. Initially we examined frequencies of total monocytes (CD66b^-^ CD3e^-^ CD19^-^ CD5^-^ gating on light scatter, gating shown in **Supplementary Figure 6A**) and found they were comparable to HC at BL (HC vs BL CIT and IBR p>0.9999) (**Figure 4A**). Importantly, frequencies of monocytes significantly increased from BL in both the CIT and IBR treatment groups. We next examined monocyte subsets: classical (CD14^+^ CD16^-^), intermediate (CD14^+^ CD16^+^), and nonclassical (CD14^-^ CD16^+^). Statistically significant increases in frequencies of all monocyte subsets were observed in both treatment groups (**Figure 4A**). It is indeed notable that this critical immune subset is so positively responsive to tumor debulking and could be viewed as a favorable modulation of the innate immune system.

We further examined changes in expression levels (using gMFI) of surface proteins indicative of monocyte functional status (**Figures 4B–D**, **Supplementary Figure 6B**). The chemokine receptor CCR2 has been implicated in monocyte and CLL B cell interactions and is primarily expressed by proinflammatory monocytes (56). Within CLL tumor microenvironments, CCR2-expressing monocytes are highly immunosuppressive (57). CCR2 expression was increased on all monocyte subsets in CIT treated patients and increased on intermediate and nonclassical monocytes in IBR treated CLL (**Figures 4B–D**, left column). CD38 expression levels on monocytes have been linked to inflammatory cytokine secretion and glycolytic activity (58). CD38 was increased on all monocyte subsets in CIT treated patients and was unchanged in IBR treated patients (**Figures 4B–D**, middle column). MHC class II expression was investigated as a surrogate marker of antigen presentation capacity and activation state (59). At BL, classical monocytes expressed lower levels of HLA-DR relative to HC (p=0.0028 for CIT and p=0.0143 for IBR cohort). For both CIT and IBR treated cohorts, HLA-DR expression was increased at RE compared to BL, approximating, or exceeding HC levels (**Figures 4B–D**, right column).

Taken together, these data show that CIT and IBR treatments dramatically increase the prevalence and functional status of monocyte subsets. However, major differences were observed between CIT and IBR treated CLL patients when functional surface proteins were analyzed at RE. CCR2, CD38, and HLA-DR surface expression were homogeneously elevated in all monocyte subsets in the CIT treated cohort at 6-month RE. Within the IBR treated cohort, changes in surface CCR2 and CD38 expression were more heterogeneous across the major monocyte subsets, however HLA-DR increased at RE in all monocyte subsets. These data are summarized in **Figure 5D**.

Dendritic cells (DCs) function as principal sensors of pathogen-associated molecular patterns (PAMPs) and damage-associated molecular patterns (DAMPs), are present in tissues and blood, connecting the innate and adaptive arms of the immune system. We evaluated DCs in both patient cohorts by comparing frequencies of total Lin^-^ HLA-DR^+^ as well as the distribution of myeloid (CD11c^+^ CD123^-^) and plasmacytoid DCs (CD11c^-^ CD123^+^) within the Lin^-^ HLA-DR^+^ gate (gating scheme shown in **Supplementary Figure 6C**). There was heterogeneity in frequencies of Lin^-^ HLA-DR^+^ cells and myeloid DC at BL in both treatment groups compared to HC (**Figure 4E**). Percentages of plasmacytoid DC were also significantly reduced at BL compared to HC as previously reported (**Figure 4E**) (60). Myeloid DC were significantly increased at RE in both CIT and IBR cohorts. We observed an increase in mDC in 7/10 CIT treated patients (p=0.0137 BL vs 6-month RE) and 17/23 IBR treated patients (p=0.0006 BL vs 24-month RE) (**Figure 4E**). We simultaneously saw an increase in prevalence of pDC in 8/10 CIT treated patients (p=0.0117 BL vs 6-month RE) and 17/23 IBR treated CLL patients (p=0.0006 BL vs 24-month RE) (**Figure 4E**). The alterations in monocyte and DC populations with the two CLL treatments are summarized in **Figure 5D**.

### Clinical outcomes in CLL treatment groups

As immune status can impact long-term outcomes in CLL patients who have an immunocompromised status even when untreated, we assessed clinical features of our study cohorts from the time of CLL diagnosis to present. The data are summarized in **Supplementary Table 2**. We observed no obvious association between immune parameters at RE with treatment type, infection history, second malignancies or long-term outcomes. Regarding survival, 4 of the 10 CIT patients are deceased, all 5 patients that received frontline IBR are living, and 12 of the 18 R/R patients are deceased. The latter is not unexpected as patients with R/R status typically do not do well with second line therapies (61–64). The 4 deceased CIT patients had unmutated IGHV status and CLL-IPI scores that ranged from 3-6. Among the deceased R/R patients, there were no obvious associations with IGVH mutation status or CLL-IPI score. We did note that 6 of the 12 deceased R/R patients were diagnosed with Rai stage III-IV disease at the time the baseline sample was collected. Additional clinical features that were considered in our study included serum immunoglobulin, infections, and second malignancies, all post-CLL diagnosis. Many patients evidenced sustained hypogammaglobulinemia, recurrent upper respiratory or genital tract infections, and secondary cancer development post CLL diagnosis. Finally, multiple patients that received SARS-COV2 vaccinations still developed COVID-19 infections, as reported (65). These findings are consistent with prior observations that historical and current treatment approaches have yet to truly overcome the impaired immune status that accompanies CLL disease (66).

## Discussion

A major conundrum in CLL treatment choice has been achieving optimal tumor burden reduction with reduced off-target toxicity of immune system components and function. Restoration of immune function, particularly of innate immune effector subsets, as they provide frontline protection against infectious agents, is critical to limit clinical complications and improve vaccine responses. With the undisputed success of novel agents, notably IBR, and the newly emerging efficacies of second and third generation BTKi derivatives with fewer off-target toxicities, CIT is almost obsolete as a treatment option for most CLL patients. However, we take note that pentostatin-based CIT is less toxic to the bone marrow than fludarabine-based CIT and thus a more suitable treatment strategy to compare to IBR (8). Here we took advantage of our ability to document the blood immune repertoire at baseline and response evaluation in pentostatin-based CIT and IBR approaches. This was done to conduct a deeper examination of these treatments to favorably restore immune function, which might be reflected with improved clinical outcomes. A summary of the immune alterations in CIT and IBR is provided in **Figure 5**.

Striking differences in therapy responses were observed in CLL B cell and T cell subsets. At the selected RE timepoints (6 months after CIT; 12-24 months after IBR initiation), both therapies as expected, largely normalized blood counts and significantly reduced CLL leukemic burden. CIT was much more effective in reducing CLL B cells, while IBR had a muted response over time in reducing CLL burden but nevertheless generated clinical responses. It is particularly noteworthy that conventional B cells did not recover in either therapy group at their respective response evaluation timepoints, consistent with persistent documented hypogammaglobulinemia (41). Interestingly, after CD19^+^ CD5^+^ exclusion, significant modulation of T cell subset frequencies was limited to Treg and CD8^+^ T cells in the IBR, but not CIT, treatment group. Reductions in frequencies of T cells expressing exhaustion markers varied, most notably after IBR. Regarding innate immune subsets, NK cell changes were more profound in CIT treated patients than IBR. Increased monocyte and DC incidence and function occurred in both treatment groups. Regardless of the improvements in the adaptive and innate immune repertoires, clinical complications and outcomes were highly similar between the CIT and IBR patient cohorts. Of primary importance, our study shows that parallel assessment of CLL B cell population kinetics with immune subset levels and function is highly informative in patients undergoing any treatment regimen. Our approach to perform a deep dive into innate and adaptive immune subset status in conjunction with assessment of clinical complications and outcomes can be a platform for future designs of treatment evaluation in CLL or other hematologic malignancies. Given our data for both cohorts, it is clear that future regimen strategies are needed to place the CLL patient immune status at a more substantial functional level, particularly with regard to restoration of conventional B cells thus, more favorably modifying clinical complications.

Changes in frequencies and expression levels of surface markers with known functions to B cell biology were examined in patients receiving CIT versus IBR. CD21^+^ and CD27^+^ CLL B cells remained unaltered or increased on CLL B cells after CIT (8/10 patients). In contrast, frequencies of CD24^+^, CXCR4^+^ and CXCR5^+^ CLL B cells showed opposite results and were decreased relative to BL after CIT. The decrease in frequencies and expression levels of CXCR4^+^ and CXCR5^+^ CLL B cells after CIT may contribute to the increased efficacy of this treatment in reduction of leukemic B cell tumor burden. Decreased frequencies of CLL B cells expressing CD21 or CD27 were observed in the IBR cohort. In CLL, increased expression of CD27 has been correlated with ZAP-70 signaling and functional capacity to interact with the microenvironment (22). Using single cell immune profiling, Rendeiro et al. also reported reduced expression of CD27 in IBR treated CLL (24). These observations of diminishing CD27 expression may be compatible with the known ability of IBR to reduce CLL B cell microenvironmental interactions. CD23 was the only surface marker examined that showed reduced frequency and expression levels with both CIT and IBR treatment. Importantly, CD23 expression correlates with CLL disease activity and tumor burden (67, 68). Regardless of the reductions in CLL tumor burden and/or phenotypic alterations with either therapy, a significant number of patients remained serum immunoglobulin deficient. This key clinical correlate likely provides much insight into the increased susceptibility to infections, including COVID-19, regardless of vaccination, documented in this study comparing two clinically effective regimens. A recent study by Noto, et al., endorsed intravenous or subcutaneous immunoglobulin replacement in CLL patients with hypogammaglobulinemia to manage increased infection risk (69). While current therapies may be efficacious in targeting leukemic B cells and restoring T, NK, monocyte, and dendritic cells, failure to reconstitute functional conventional B cells and humoral immunity, will maintain the immunocompromised state in CLL patients (3, 10).

A significant difference between the CIT and IBR cohorts uncovered in this study was T cell exhaustion status. The reductions in Treg frequency along with percentages of T cells expressing the exhaustion markers PD-1 and TIGIT in IBR treated patients concur with previous reports (44, 46, 70). This pattern with IBR treatment, which includes decreased frequencies in TIM3^+^ CD4^+^ and CD8^+^ T cells, is in striking contrast to our findings that CIT had no effect on reduction of PD-1, TIGIT or TIM3 expressing T cells. Indeed, the general trend for CIT treatment was increased frequencies of T cells expressing PD-1, TIGIT and CTLA-4. We also found increased PD-1 frequencies in residual leukemic CLL B cells in a subset of CIT treated patients. This finding has implications for the use of PD-1 checkpoint inhibitors that may block the activity of PD-1 present on the surface of cells in these patients post CIT treatment (71). An enlightening observation between CIT and IBR treatment is decreased T cell exhaustion by IBR, whereas CIT induced the reverse at the evaluation timepoint examined. This finding also reinforces that the current switch from CIT to novel agent-based therapy for all CLL patients can be justified on an immune enhancement basis as well.

We also examined modulation of innate immune subsets in response to CIT and IBR treatment where we found the most overall consistent trend. Here we found, regardless of therapy approach, that this increased innate immune subset frequencies. Total NK cells after treatment were increased or comparable in frequency to HC at BL. Patients receiving CIT had increased frequencies of total NK cells while those treated with IBR showed more heterogeneous responses, in accord with previous observations (25, 72). It should be noted that some NK cell functional characteristics that have been described as dysfunctional were attributed to leukemic escape mechanisms and not due to intrinsic NK cell dysfunction (73). Monocyte subsets increased in frequency in both treatment groups, although they were more significantly elevated in the CIT cohort. Changes in expression of markers indicative of heightened monocyte function including CCR2, CD38 and HLA-DR were also compared between the two treatment groups. CIT treatment increased expression levels of all three functional markers on all monocyte subsets. In contrast, only HLA-DR was significantly increased on all monocyte subsets in IBR treated patients. We observed a similar response pattern of increased frequencies of myeloid and plasmacytoid DCs in both patient groups. These findings suggest that some CIT approaches will be beneficial to CLL patients if enhanced monocyte and DC function impact their health and clinical outcomes. However, in this study, IBR therapy also generated substantial increases in innate subset frequencies, which we propose is an encouraging rationale for this approach in CLL.

In summary, this study parsed out major findings for pentostatin-based CIT and IBR treatment that integrated immune therapy induced immune repertoire changes with clinical features. These findings highlight the importance of a full and comprehensive assessment of leukemic B cell and the global immune repertoire as we continue with the evolution of novel agent treatment strategies to treat B cell malignancies.

## Data availability statement

The original contributions presented in the study are included in the article/**Supplementary Material**. Further inquiries can be directed to the corresponding author.

## Ethics statement

The studies involving humans were approved by Mayo Clinic Human Subjects Institutional Review Board. The studies were conducted in accordance with the local legislation and institutional requirements. The human samples used in this study were acquired from primarily isolated as part of your previous study for which ethical approval was obtained. Written informed consent for participation was not required from the participants or the participants’ legal guardians/next of kin in accordance with the national legislation and institutional requirements.

## Author contributions

BW: Conceptualization, Validation, Writing – original draft, Writing – review & editing, Data curation, Formal Analysis, Investigation, Methodology. BM: Conceptualization, Data curation, Formal Analysis, Investigation, Methodology, Validation, Writing – original draft, Writing – review & editing. KG: Data curation, Investigation, Writing – review & editing. PL: Data curation, Investigation, Writing – review & editing. SP: Data curation, Writing – review & editing, Conceptualization, Supervision, Validation. NK: Conceptualization, Data curation, Supervision, Validation, Writing – review & editing. KM: Conceptualization, Supervision, Validation, Writing – review & editing, Funding acquisition, Resources, Writing – original draft.
