# Supplementary material for: Comparison of the blood immune repertoire with clinical features in chronic lymphocytic leukemia patients treated with chemoimmunotherapy or ibrutinib
Source: Front Oncol. 2023 Dec 4;13:1302038. doi: 10.3389/fonc.2023.1302038 (PMC10725910; doi:10.3389/fonc.2023.1302038)
Supplement: Supplementary file 1 [file DataSheet_1.docx]

Supplementary Material

# Supplementary Data

# Supplemental Figure 1. CIT and IBR treatments resolve pre-treatment cytopenias

# Supplemental Figure 2. Flow cytometry gating strategy for B cell subsets

# Supplemental Figure 3. B cell supplementary data

# Supplemental Figure 4. Flow cytometry gating strategy for T cell subsets

# Supplemental Figure 5. Flow cytometry gating strategy for NK cell subsets

# Supplemental Figure 6. Flow cytometry gating strategy for monocyte and DC subsets

# Supplemental Table 1. Flow cytometry panel antibodies and reagents.

# Supplemental Table 2. CLL patient clinical serum Ig status and immunization/infection/cancer history


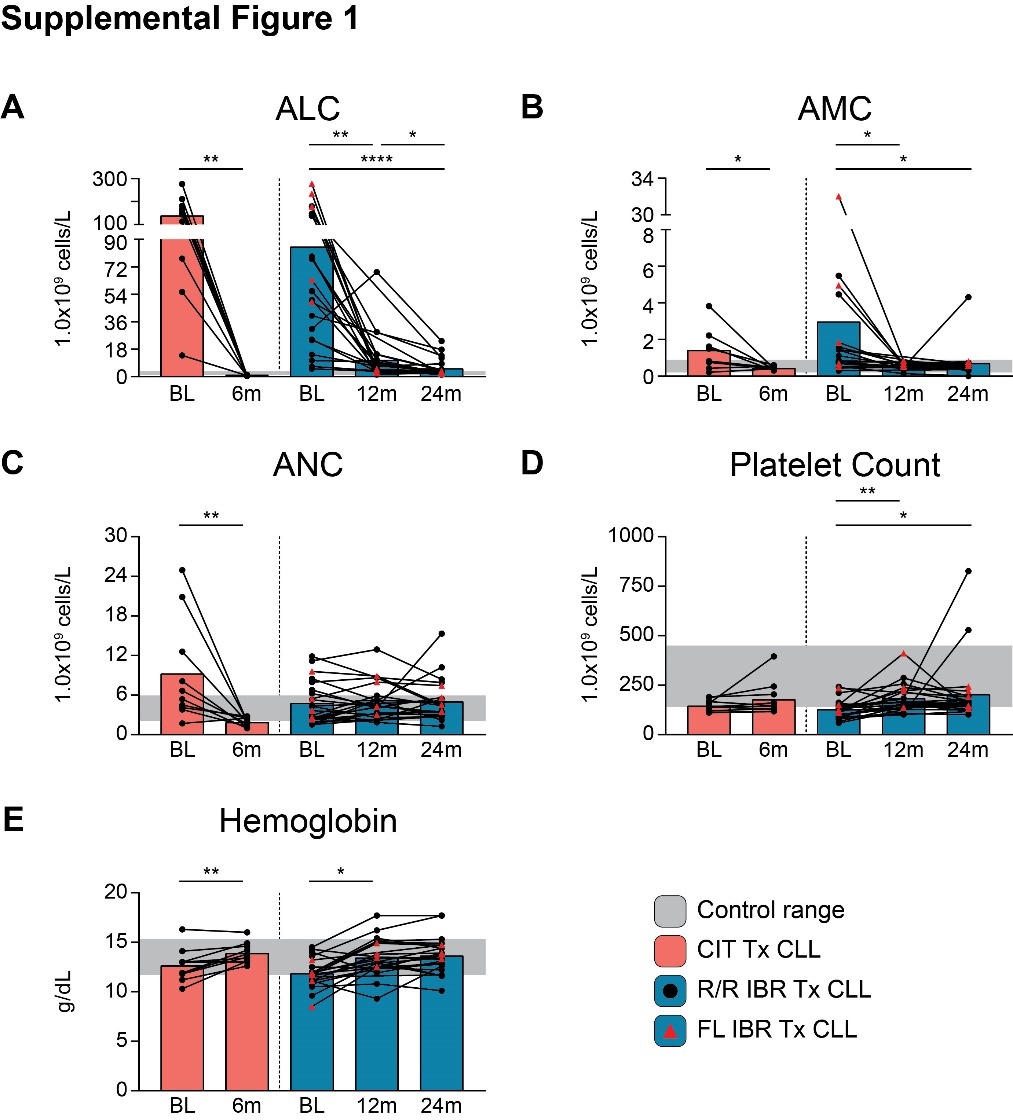


**Supplementary Figure 1.** **(A-E)** Whole blood CBC were done on CLL patients at the indicated timepoints. The AMC parameter was not obtained for 4 CLL patients (CIT patients: CLL 1, CLL 7, and relapsed CLL on IBR: CLL 27 and CLL 33). Gray region indicates the healthy control range obtained from Stem Cell Technologies Frequencies of Cell Types in Human Peripheral Blood (DOCUMENT #23629 | VERSION 4.1.0). ALC 1.1-3.5x10^9^ cells/L, AMC 0.2-0.9x10^9^ cells/L, ANC 2.09-5.97x10^9^ cells/L, Platelet count 140-450x10^9^ cells/L, Hemoglobin 11.7-15.3 g/dL. Statistical significance shown are as follows *p<0.05, **p<0.01, ***p<0.001, and ****p<0.0001.


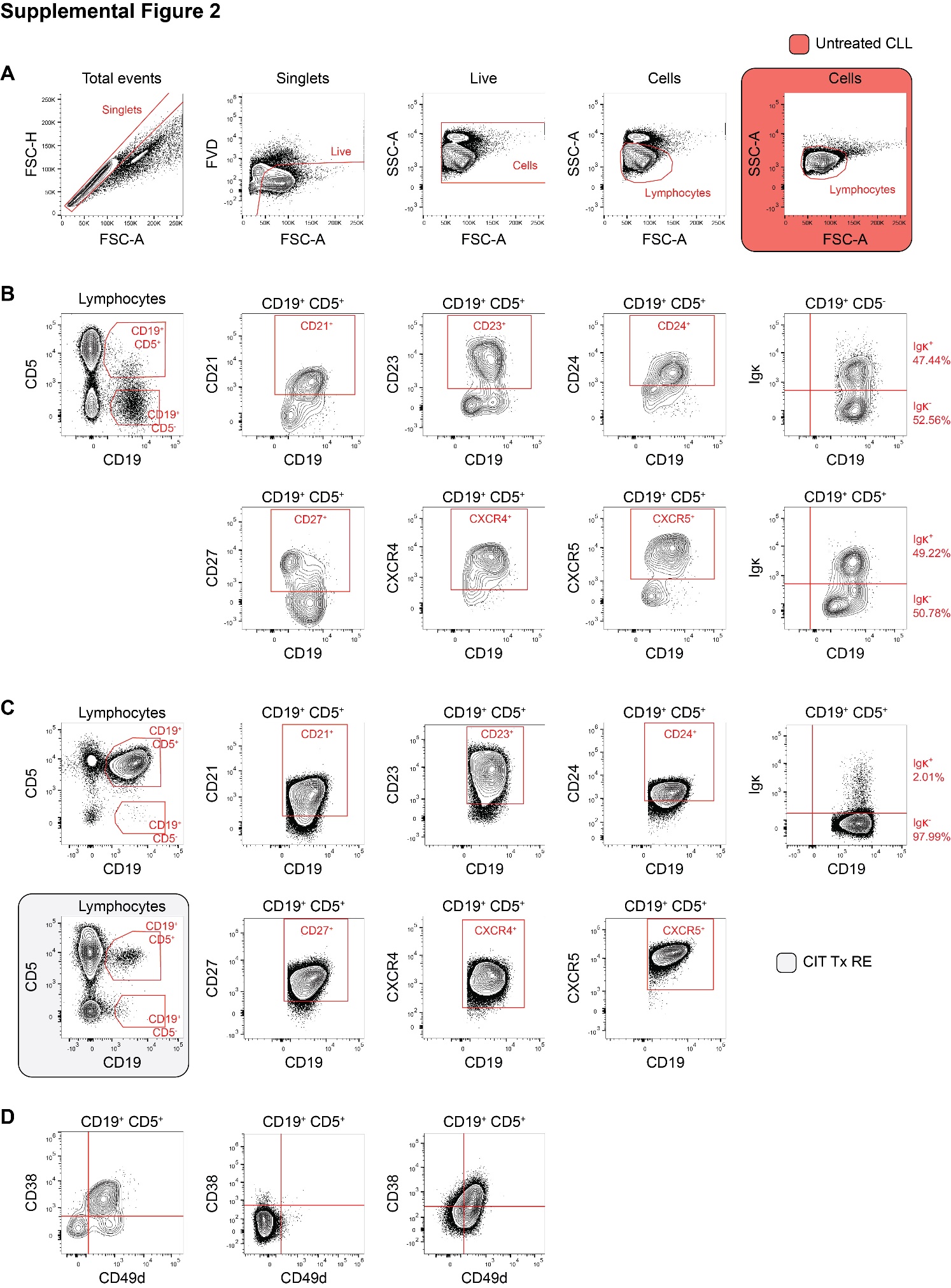


**Supplementary Figure 2.** **(A)** Sequential gating strategy for “Singlets”, “Live”, “Cells”, and “Lymphocytes” from a representative HC. An untreated CLL patient FSC-A by SSC-A plot is shown for the “Cells” gate is specified in magenta. **(B)** Sequential gating strategy specifying the CD19^+^ CD5^-^ and CD19^+^ CD5^+^ populations from a representative HC. Adjacent are the gates for CD21^+^, CD23^+^, CD24^+^, CD27^+^, CXCR4^+^, CXCR5^+^, and Igк^+^ CD19^+^ CD5^+^ cells. Top row on far right shows the polyclonal light chain Igк^+^ distribution for CD19^+^ CD5^-^ cells. **(C)** Sequential gating strategy specifying the CD19^+^ CD5^-^ and CD19^+^ CD5^+^ populations from a representative untreated CLL patient. Adjacent are the gates for CD21^+^, CD23^+^, CD24^+^, CD27^+^, CXCR4^+^, CXCR5^+^, and Igк^+^ CD19^+^ CD5^+^ cells. Shaded in a gray box is the same patient at 6-month response evaluation post-CIT treatment. **(D)** Left plot shows CD19^+^ CD5^+^ cells from a representative HC. Middle plot shows CD19^+^ CD5^+^ cells from a representative untreated CLL patient with a negative CD38 and CD49d status. Right plot shows CD19^+^ CD5^+^ from a representative untreated CLL patient with a positive CD38 and CD49d status.


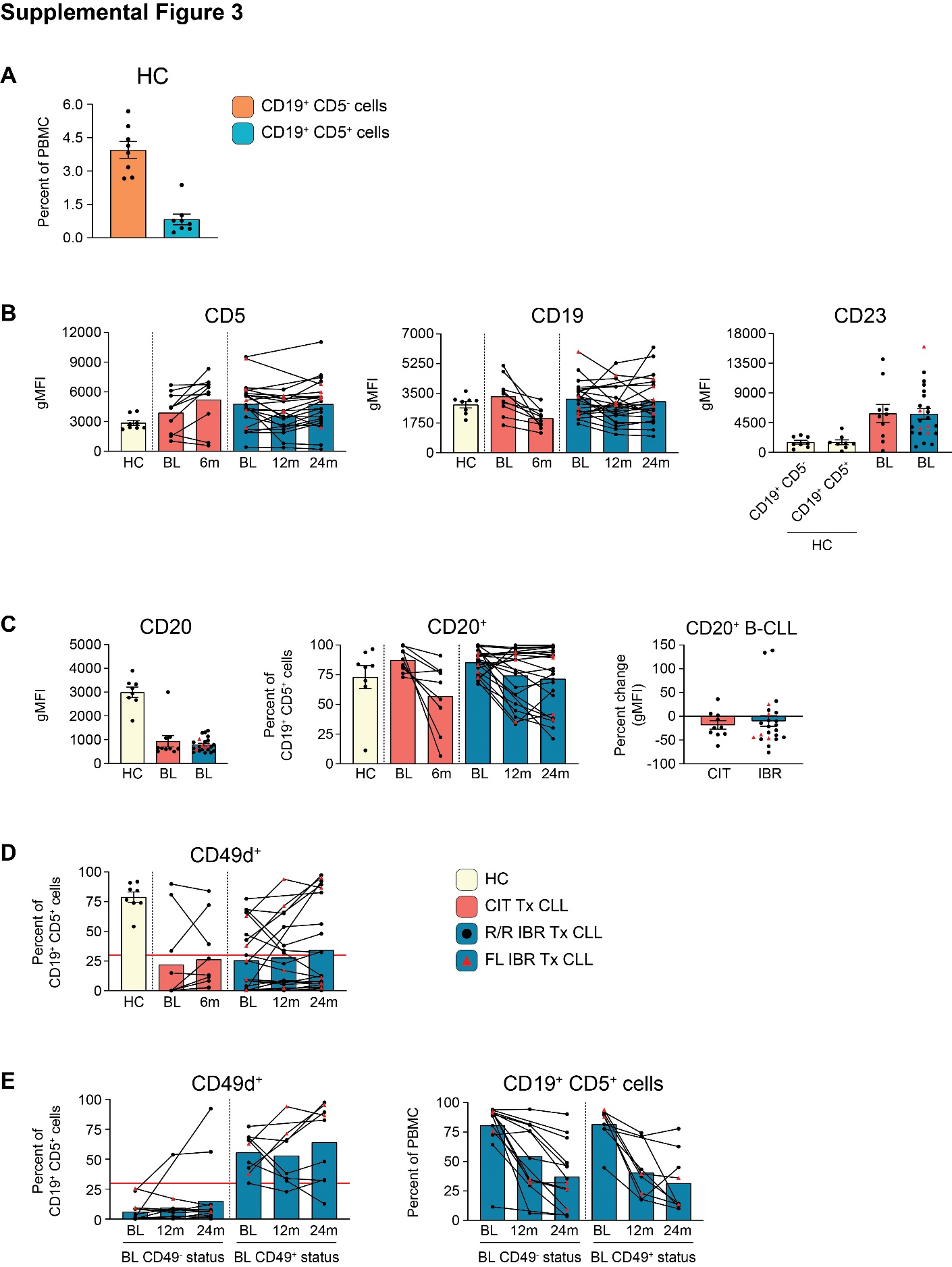


**Supplementary Figure 3.** **(A)** Frequency of CD19^+^ CD5^-^ and CD19^+^ CD5^+^ cells are reported as a percent of PBMC from HC. **(B)** Geometric mean fluorescence intensity (gMFI) for surface CD5 and CD19 on CD19^+^ CD5^+^ cells are shown. gMFI is shown for CD23 on CD19^+^ CD5^-^ cells from HC and CD19^+^ CD5^+^ from HC and CLL patients at BL. **(C)** On left, the gMFI of CD20 is shown for CD19^+^ CD5^+^ CD20^+^ cells from HC and CLL patients at BL. In middle, the frequency of CD20^+^ cells as a percent of CD19^+^ CD5^+^ cells from HC and CLL patients. On right, the percent change in CD20 gMFI on CD19^+^ CD5^+^ CD20^+^ cells from CIT and IBR treated patients between BL and RE (24-month RE for IBR treated patients). **(D)** The frequency of CD49d^+^ cells as a percent of CD19^+^ CD5^+^ cells from HC and CLL patients. The red line is set at the y-axis=30% which is the clinical cutoff for designating a CD49d positive status for CLL cells. **(E)** On left, IBR treated CLL patients were divided by their BL CD49d status. On right, the frequency of CD19^+^ CD5^+^ CLL cells as a percent of PBMC is shown upon IBR treatment.


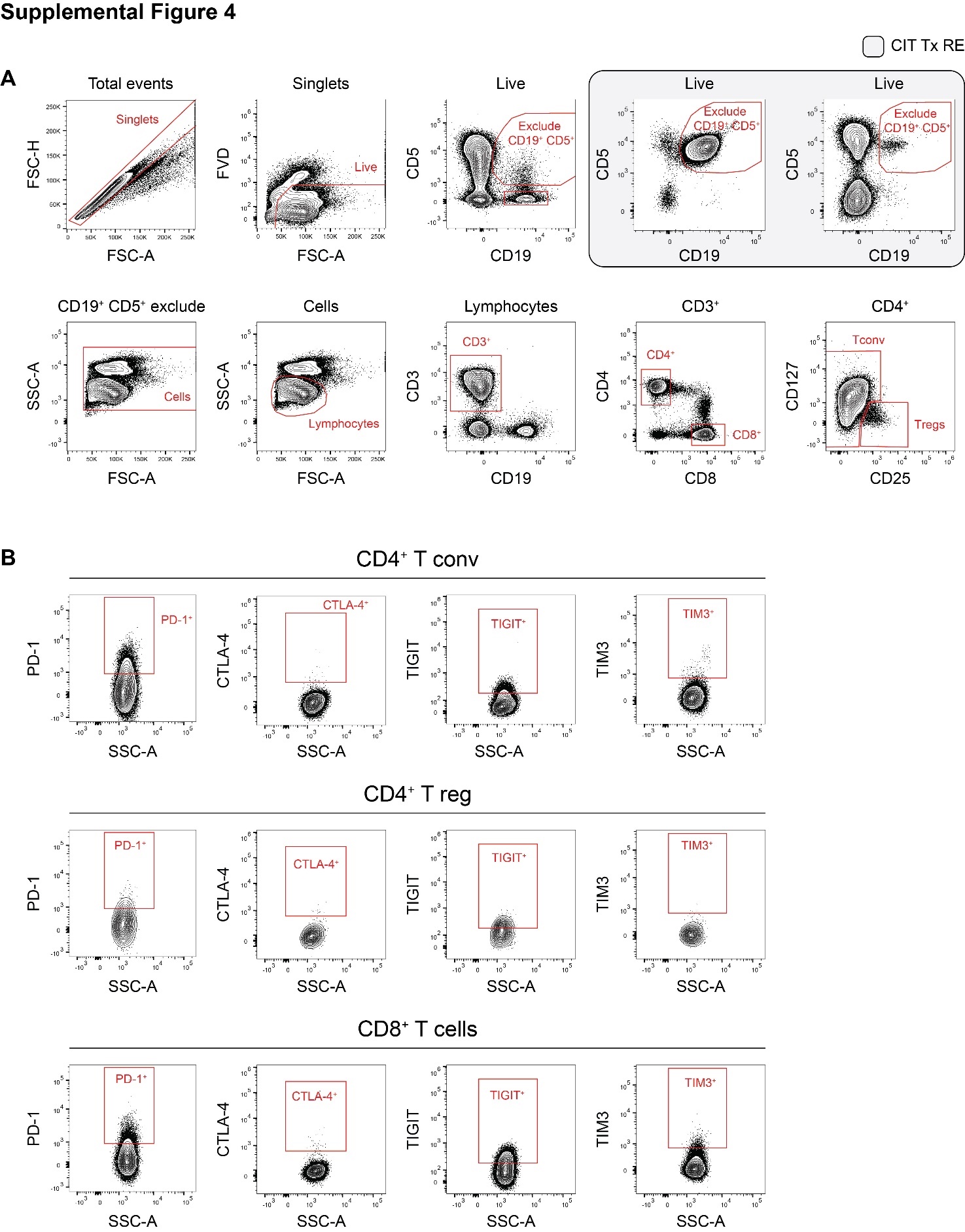


**Supplementary Figure 4.** **(A)** T cell panel gating strategy is shown for a representative HC. Shaded in a gray box are the plots showing the CD19^+^ CD5^+^ exclusion gate from an untreated CLL patient at baseline (left) prior to CIT treatment at 6-month response evaluation (right). **(B)** HC representative gates used for specifying PD-1^+^, CTLA-4^+^, TIGIT^+^, and TIM3^+^ CD4^+^ Tconv, CD4^+^ Treg, and CD8^+^ T cells.


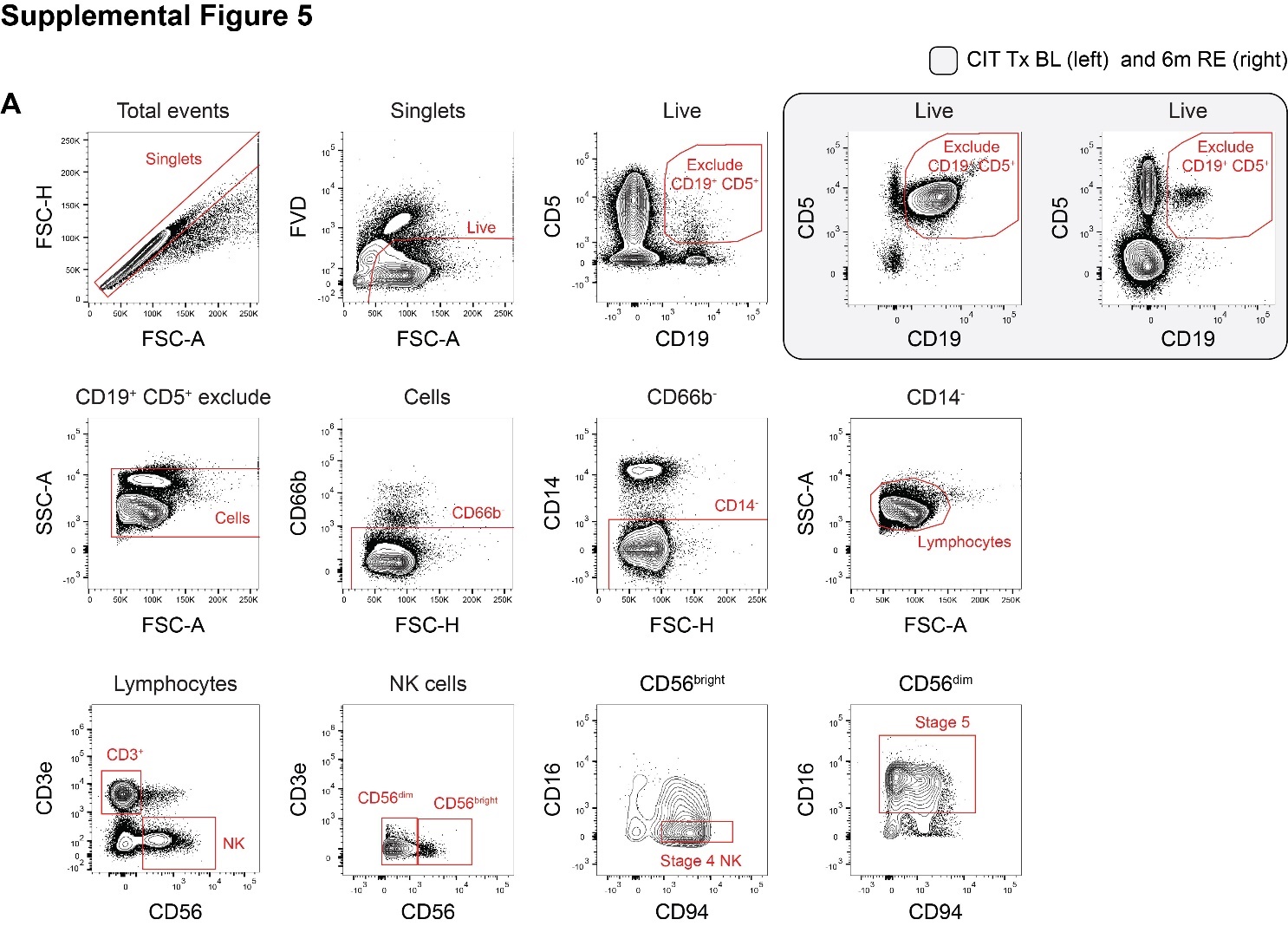


**Supplementary Figure 5.** **(A)** NK cell gating tree shown for a representative HC. Shaded in a gray box are the plots showing the CD19^+^ CD5^+^ exclusion gate from an untreated CLL patient at baseline (left) prior to CIT treatment at 6-month response evaluation (right).


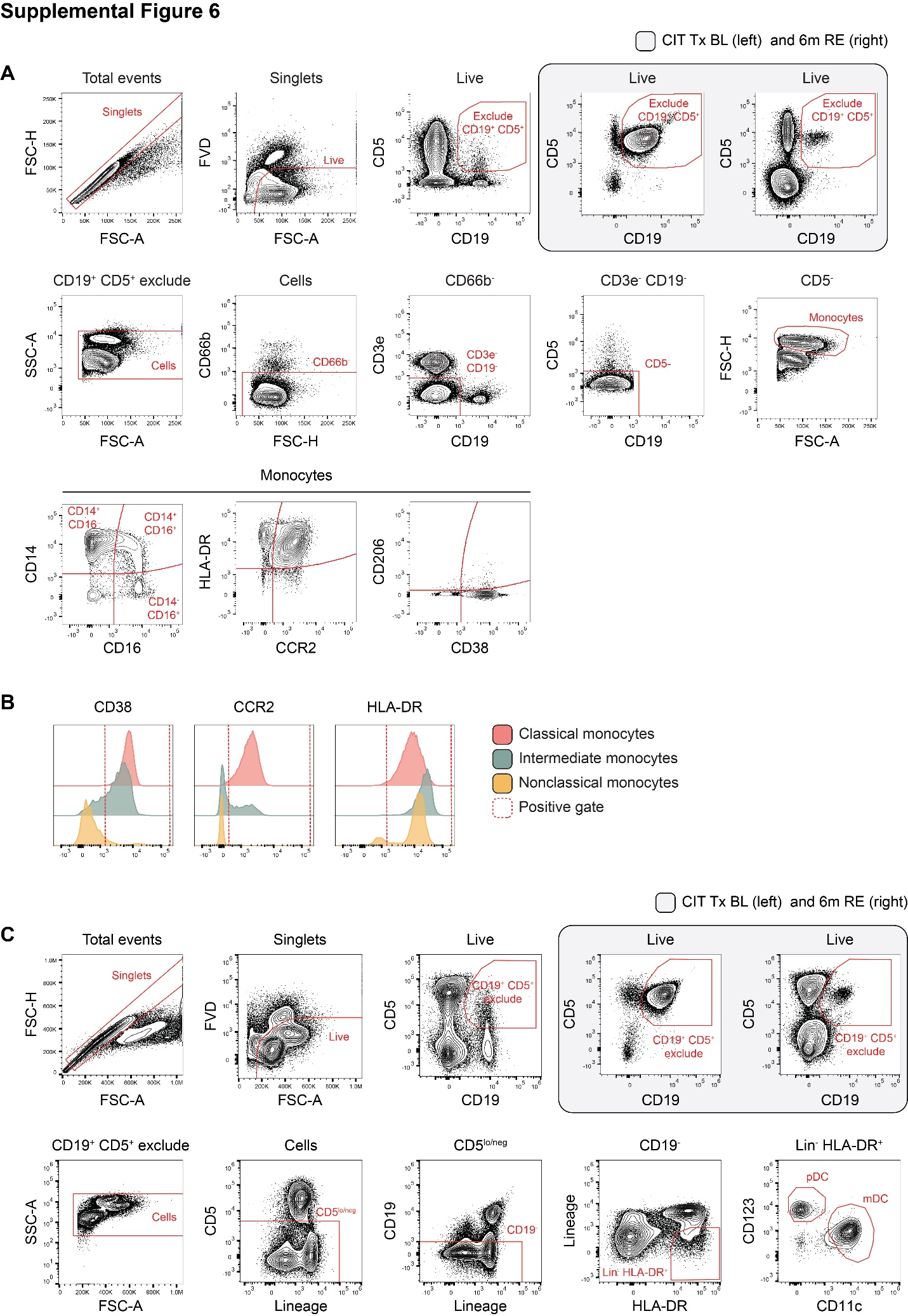


**Supplementary Figure 6.** **(A)** Monocyte gating tree shown for a representative HC. Shaded in a gray box are the plots showing the CD19^+^ CD5^+^ exclusion gate from an untreated CLL patient at baseline (left) prior to CIT treatment at 6-month response evaluation (right). Outlined below are the plots showing the quadrant gates used to specify classical (CD14^+^ CD16^-^), intermediate (CD14^+^ CD16^+^), and non-classical (CD14^-^ CD16^+^) monocytes. In addition, quadrant gates were used to specify HLA-DR^+^, CCR2^+^, and CD38^+^ monocytes. These gates were subsequently applied to classical, intermediate, and non-classical monocyte subsets. **(B)** Histograms showing the distribution of each indicated surface protein expression by monocyte subset (cell counts are normalized to mode for each monocyte subset). Red lines designate the bounds of positivity when reporting the gMFI of indicated surface protein expressing monocyte subsets shown in **Fig. 4B-D** (i.e., gMFI of surface CD38 expression on CD38^+^ intermediate monocytes is reported). **(C)** Dendritic cell (DC) gating tree shown for a representative HC. Shaded in a gray box are the plots showing the CD19^+^ CD5^+^ exclusion gate from an untreated CLL patient at baseline (left) prior to CIT treatment at 6-month response evaluation (right).

**Supplemental Table 1**

| Panel | Marker | Fluorochrome | Clone | Vendor | Cat. No. |
| --- | --- | --- | --- | --- | --- |
| Dendritic cell | CD1c | BV421 | F10/21A3 | BD Biosciences | 565050 |
|  | CD5 | PE/Cy7 | L17F12 | Biolegend | 364008 |
|  | CD11c | FITC | B-ly6 | BD Biosciences | 561355 |
|  | CD19 | APC/Fire 750 | SJ25C1 | Biolegend | 363030 |
|  | CD123 | BV605 | 6H6 | Biolegend | 306026 |
|  | CD141 | APC | 1A4 | BD Biosciences | 564123 |
|  | HLA-DR | BV786 | G46-6 | BD Biosciences | 564041 |
|  | CD3 | PerCP | SK7 | BD Biosciences | 347344 |
|  | CD14 | PerCP | MφP9 | BD Biosciences | 340660 |
|  | CD20 | PerCP | 2H7 | Biolegend | 302324 |
|  | CD56 | PerCP | HCD56 | Biolegend | 318342 |
| NK/Monocyte | CD3e | FITC | OKT3 | Biolegend | 317306 |
|  | CD5 | BV605 | L17F12 | BD Biosciences | 742550 |
|  | CD14 | BV650 | M5E2 | Biolegend | 301836 |
|  | CD16 | BUV496 | 3G8 | BD Biosciences | 564653 |
|  | CD19 | APC/Fire 750 | SJ25C1 | Biolegend | 363030 |
|  | CD38 | BUV737 | HB7 | BD Biosciences | 564686 |
|  | CD56 | PE/Cy7 | MEM-188 | Biolegend | 304628 |
|  | CD66b | V450 | G10F5 | BD Biosciences | 561649 |
|  | CD94 | BUV395 | HP-3D9 | BD Biosciences | 743954 |
|  | CD163 | PE | GHI/61 | Biolegend | 333606 |
|  | CD206 | PE/Dazzle | 15-2 | Biolegend | 321130 |
|  | CCR2 | BV785 | K036C2 | Biolegend | 357234 |
|  | HLA-DR | Alexa Fluor 700 | L243 | Biolegend | 307626 |
| B cell | CD5 | BV605 | L17F12 | BD Biosciences | 742550 |
|  | CD19 | APC/Fire 750 | SJ25C1 | Biolegend | 363030 |
|  | CD20 | BUV395 | 2H7 | BD Biosciences | 563782 |
|  | CD21 | BV785 | B-ly4 | BD Biosciences | 740969 |
|  | CD23 | BUV737 | M-L233 | BD Biosciences | 741853 |
|  | CD24 | FITC | ML5 | Biolegend | 311104 |
|  | CD27 | Alexa Fluor 700 | M-T271 | Biolegend | 356416 |
|  | CD38 | BUV496 | HIT2 | BD Biosciences | 564657 |
|  | CD49d | PE | L25 | BD Biosciences | 340296 |
|  | CXCR4 | PE/Dazzle | 12G5 | Biolegend | 306526 |
|  | CXCR5 | Alexa Fluor 647 | RF8B2 | BD Biosciences | 558113 |
|  | Ig kappa | PE/Cy7 | MHK-49 | Biolegend | 316520 |
|  | IgM | BV650 | G20-127 | BD Biosciences | 740595 |
| T cell | CD3 | BV785 | UCHT1 | Biolegend | 300472 |
|  | CD4 | PE/Cy7 | RPA-T4 | Biolegend | 300512 |
|  | CD5 | BV605 | L17F12 | BD Biosciences | 742550 |
|  | CD8 | BUV496 | RPA-T8 | BD Biosciences | 612942 |
|  | CD19 | APC/Fire 750 | SJ25C1 | Biolegend | 363030 |
|  | CD25 | BUV395 | M-A251 | BD Biosciences | 740290 |
|  | CD38 | BUV737 | HB7 | BD Biosciences | 564686 |
|  | CD127 | Alexa Fluor 700 | A019D5 | Biolegend | 351344 |
|  | CTLA-4 | BV421 | BNI3 | Biolegend | 369606 |
|  | PD-1 | BV650 | EH12.2H7 | Biolegend | 329950 |
|  | TIGIT | FITC | MBSA43 | Invitrogen, eBioscience | 11-9500-42 |
|  | TIM3 | APC | F38-2E2 | Biolegend | 345012 |
| All panels | Fixable Viability Stain 510 | - | - | BD Biosciences | 564406 |

**Supplemental Table 2: CLL patient clinical serum Ig status and immunization/infection/cancer history**

|  | Patient ID | CLL diagnosis | BL flow date | Serum Ig date(s) | IgG^a^ | IgM^b^ | IgA^c^ | Infections; Date Post CLL diagnosis | Other cancers; Date Post CLL diagnosis | Living status |
| --- | --- | --- | --- | --- | --- | --- | --- | --- | --- | --- |
| CIT Tx | CLL 1 | Nov-2005 | Jan-2011 | Mar-2016 | 844 | ND | ND | Coccidioidomycosis; Mar-2016 |  | Live |
|  | CLL 2 | Aug-2010 | Jan-2011 | May-2016  May-2022 | 1060  427 | 47  ND | 77  ND | Chronic sinusitis; Sep-2016  Urinary tract infection; May-2022 | Basal cell (scalp); Jun-2016 | Deceased |
|  | CLL 3 | Mar-2009 | Oct-2011 | Oct-2012  Apr_2023 | 390  602 | 14  ND | 48  ND |  |  | Live |
|  | CLL 4 | Before 2012 | Jan-2012 | Mar-2013 | 374 | 21 | 39 | Urinary tract infection; Jun-2013  Acute sinusitis; Oct-2013 |  | Live |
|  | CLL 5 | Jan-2009 | Feb-2012 | Feb-2012  Apr-2023 post-IVIG* | 656  705* | 23  ND | 65  ND |  | Prostate; Jan-2021 | Live |
|  | CLL 6 | Jun-2001 | May-2012 | Aug-2020 | 735 | 21 | 133 |  |  | Live |
|  | CLL 7 | Unknown | May-2012 | Sept-2019  Mar-2021 | 359  411 | 6  <5 | 35  41 | Esophagitis candida; Aug-2019  Urinary tract infection; Oct-2020 | Basal cell (skin); Oct-2013 | Deceased |
|  | CLL 8 | Apr-2006 | Aug-2012 | Oct-2018  Mar-2023 | 508  590 | 9  <10 | 45  32 | Recurrent sinusitis; Oct-2018 |  | Live |
|  | CLL 9 | Oct-2008 | Sep-2012 | Jun-2017 | 869 | 12 | 123 | COVID-19 pneumonia; Sep-2022  Acute cystitis; Oct-2022 |  | Deceased |
|  | CLL 10 | May-2008 | Nov-2012 | Nov-2012  Jan-2014  Aug-2021 | 714  783  564 | 21  30  ND | 87  92  ND |  | Prostate/Thyroid; Apr-2014  Gastric; May-2021 | Deceased |
| FL IBR Tx | CLL 11 | Apr-2014 | May-2014 | Jun-2023 | 440 | 9 | 20 | Acute cystitis; Jun-2023 |  | Live |
|  | CLL 12 | Mar-2014 | Mar-2016 | Apr-2014 | 472 | 25 | 67 |  |  | Live |
|  | CLL 13 | Jan-2014 | Jan-2016 | Sept-2021 | 530 | ND | ND | COVID-19; Oct-2021  (SARS-COV2 VAC Aug-2021) |  | Live |
|  | CLL 14 | Nov-2016 | Nov-2016 | Sept-2021 | 802 | ND | ND | COVID-19; Dec-2021  (SARS-COV2 VAC Aug-2021) | Basal cell; Squamous cell; Melanoma skin; historical | Live |
|  | CLL 15 | Feb-2015 | Dec-2016 | Dec-2016 | 388 | 7 | 61 |  |  | Live |
| R/R IBR Tx | CLL 16 | Jun-1999 | Dec-2013 | May-2019 | 741 | 66 | 28 |  | GI; Sep-2017  Large cell lymphoma; Aug-2017 | Deceased |
|  | CLL 17 | Dec-2007 | Jan-2014 | Oct-2017 | 214 | ND | ND | Cellulitis; Oct-2018 | Breast; Jan-2018 | Deceased |
|  | CLL 18 | Jun-2001 | Apr-2014 | Apr-2014 | 909 | 96 | 13 |  | Kidney; N/A | Live |
|  | CLL 19 | Jul-2010 | Jun-2014 | Mar-2017 | 928 | <5 | 39 |  | Merkel cell; Apr-2017 | Deceased |
|  | CLL 20 | Jun-2005 | Jan-2015 | Feb-2021 | 291 | ND | ND | COVID-19 pneumonia; Mar-2021  (SARS-COV2 VAC Feb-2021) | Lung; May-2017  Squamous cell; Jul-2011  Squamous cell carcinoma (ear); Nov-2015 | Deceased |
|  | CLL 21 | N/A | Jan-2015 | Nov-2020 | 568 | <5 | 33 | COVID-19 pneumonia; Nov-2020 |  | Deceased |
|  | CLL 22 | Apr-2005 | Jan-2015 | Jan-2015  Feb-2023 | 371  464 | ND  16 | ND  13 | Chronic bronchitis; Apr-2023 |  | Live |
|  | CLL 23 | Mar-2012 | Mar-2015 | Jul-2015  Oct-2017 | 445  761 | 20  92 | 36  155 |  | Head/neck; Jul-2017 | Deceased |
|  | CLL 24 | Jun-2009 | Mar-2015 | Jul-2013  Jan-2023 | 671  324 | 56  ND | 32  ND |  |  | Live |
|  | CLL 25 | Oct-2005 | Apr-2015 | May-2015  Jan-2017 | 594  552 | <5  ND | 27  42 | Pneumonia; Mar-2019 |  | Live |
|  | CLL 26 | Oct-2007 | Aug-2015 | Aug-2015 | 108 | <5 | 3 |  |  | Deceased |
|  | CLL 27 | Mar-2013 | Feb-2016 | Nov-2022 | 618 | 117 | 1 | Sinusitis; Jul-2022  Bronchiolitis; Oct-2022 |  | Deceased |
|  | CLL 28 | Nov-2004 | Dec-2016 | Jan-2017 | 281 | ND | ND |  | Extramedullary plasmacytoma; Mar-2017  Peritoneal carcinomatosis; Aug-2021 | Deceased |
|  | CLL 29 | Aug-2013 | Apr-2014 | Mar-2023 | 671 | ND | ND |  |  | Live |
|  | CLL 30 | Nov-2006 | Feb-2015 | Oct-2013 | 838 | 8 | 39 | Condyloma wart; Aug-2016 | Lymph node/lung/bladder; Aug-2016 | Deceased |
|  | CLL 31 | Jul-2001 | Apr-2015 | Mar-2017 | 804 | ND | ND |  | Nasopharynx; Jul-2017  Brain; Jun-2019 | Deceased |
|  | CLL 32 | Jan-2009 | Aug-2015 | Feb-2018 | 1590 | 38 | 104 | Urinary tract infection; Sep-2019 |  | Live |
|  | CLL 33 | Feb-2006 | Mar-2017 | Dec-2019 | 718 | 25 | 32 | Cellulitis; Mar-2019 | T cell cutaneous lymphoma; Oct-2019  Myelodysplastic myeloproliferative overlap; Jun-2019 | Deceased |

ND indicates “Not determined” for all columns. (a) Values are in mg/dL and the normal IgG range is 767-1590 mg/dL. (b) Values are in mg/dL and the normal IgM range is 37-286 mg/dL. (c) Values are in mg/dL and the normal IgA range is 61-356 mg/dL.
